# Dissipation kinetics and biological degradation by yeast and dietary risk assessment of fluxapyroxad in apples

**DOI:** 10.1038/s41598-020-78177-6

**Published:** 2020-12-03

**Authors:** Magdalena Podbielska, Paulina Książek, Ewa Szpyrka

**Affiliations:** Department of Biotechnology, Institute of Biology and Biotechnology, University of Rzeszów, Pigonia 1, 35-310 Rzeszów, Poland

**Keywords:** Environmental sciences, Environmental chemistry

## Abstract

The aim of this study was to investigate the dissipation kinetics of fluxapyroxad in apples, the influence of biological treatment with yeast, and the estimation of dietary exposure for consumers, both adults and children. The gas chromatography technique with the electron capture detector was used to analyse the fluxapyroxad residues. Samples of apples were prepared by the quick, easy, cheap, effective, rugged and safe (QuEChERS) method. The average fluxapyroxad recoveries in apple samples ranged from 107.9 to 118.4%, the relative standard deviations ranged from 4.2 to 4.7%, and the limit of quantification was 0.005 mg/kg. The dissipation half-lives in Gala and Idared varieties were 8.9 and 9.0 days, respectively. Degradation levels of the tested active substance after application of yeast included in a biological preparation Myco-Sin were 59.9% for Gala and 43.8% for Idared. The estimated dietary risk for fluxapyroxad in apples was on the acceptable safety level (below 9.8% for children and 1.9% for adults) and does not pose a danger to the health of consumers.

## Introduction

The global drive to increase the agricultural productivity has led to the extensive use of pesticides^1^. Their use is indispensable in the modern agriculture, and they have become considered essential for ensuring the supply of good quality food. Pesticides help to improve and maintain crop quality, and are widely used to protect crops against harmful biotic factors, such as insect, pathogens, and weed^2^.

Apples are one of the most popular and most consumed fruit in the world, due to their availability throughout the year^3^. There are a rich source of phytochemicals, and have been found to have a very strong antioxidant activity, which allows them to inhibit cancer cell proliferation, inhibit lipid oxidation, and lower cholesterol on ingestion^4^. Apples, due to their high health-promoting properties, are introduced as the first component of the children’ diet.

**Figure 1:**
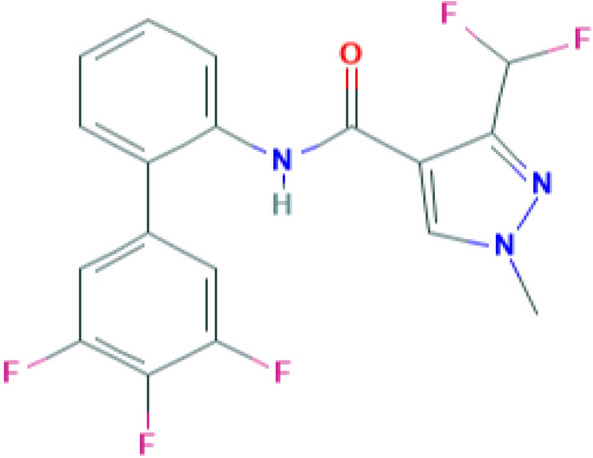
Structure of fluxapyroxad^6^.

Recently, some fungicides have been registered and widely used in agricultural production for plant disease control, which have special mode of action and diverse biological activities^5^. One of them is fluxapyroxad (IUPAC name: 3-(difluoromethyl)-1-methyl-*N*-(3′,4′,5′-trifluorobiphenyl-2-yl)pyrazole-4-carboxamide), a new systemic carboxamide fungicide developed by BASF Corporation (Fig. 1).

Fluxapyroxad has preventive properties, and it is widely used in plant protection to control a broad spectrum of fungal diseases in a wide range of crops. Fluxapyroxad is an inhibitor of the enzyme succinate dehydrogenase inhibitor (SDHI) in the complex II of the mitochondrial respiratory chain. It inhibits spore germination, and germ tube and mycelia growth in target fungi^7–9^.

Studies have shown that fluxapyroxad is highly active against several major plant pathogens, including fungal species *Ascomycete, Basidiomycete, Deuteromycota and Zygomycota*^10^*.* It is effective in a wide variety of crops, including cereals, corn, soybean, fruiting vegetables, tuberous and corm vegetables, pome fruits, and stone fruit^11^.

Currently, fluxapyroxad is being registered or introduced worldwide for use on the market. Therefore, research on its behaviour in the environment is necessary. Only a few studies have been conducted to evaluate the environmental fate, dissipation and degradation of this new fungicide. Only He et al.^12^ and Noh et al.^2^ conducted studies of fluxapyroxad in apples and soil and on the leaves of a perilla grown in a greenhouse (*Perilla frutescens var. Japonica Hara*), respectively*.* To our knowledge, the influence of microorganisms on degradation of fluxapyroxad has not been previously studied.

Changes in the concentration of active substances of plant protection products are determined by the dissipation kinetics. It is influenced by many factors, including temperature, humidity, application time, light, pH, structure and chemical properties of active substances, and the crop growth status. An important factor affecting on the pesticide degradation are microorganisms living in the environment and/or intentionally introduced into it. The microbial degradation is an important dissipation process. For this reason, the pesticide degradation by bacteria, fungi and yeast has received much attention^13^. In last decade, the Integrated Pest Management (IPM) was introduced in the EU (European Union). In IPM, biological methods are used primarily for plant protection and harvest protection during storage. In this system, the use of biological preparations containing microorganisms is essential.

The aim of this study was to determine the dissipation kinetics of fluxapyroxad in apples. A particular attention was paid to the requirements for apples intended for consumption by children. We established Pre-harvest Interval (PHI) to obtain ripe apples containing pesticide residues at levels safe for children (below 0.01 mg/kg), and estimated chronic dietary exposure to human health on the basis of amounts of fluxapyroxad from the supervised trials. The second objective of this study was to investigate in the field conditions whether the application the biological preparation Myco-Sin according to IMP may result in the faster degradation of fluxapyroxad.

## Results and discussion

### Method validation

The method was validated for evaluation of the following parameters: linearity (expressed as a coefficient of determination, R), limit of quantification (LOQ), limit of detection (LOD), matrix effect, specificity, average recovery to represent accuracy, precision (expressed as relative standard deviations RSDr), precision for within-laboratory reproducibility (expressed as RSDwr), robustness and retention time according to the European Union guideline SANTE^14^. The acceptance criteria for the method were as follows: average recoveries for the tested active substance in the range of 70–120%, precision expressed as RSD ≤ 20%, and linearity of the detector response determined by the correlation coefficient R ≥ 0.99.

Blank samples of apples were analyzed to verify the absence of interfering substances at about the retention time of the analyte. The parameters of the linear equations: slope intercept, their standard deviations and the correlation coefficient (R) were calculated. The linearity of the method was determined in triplicate at six concentrations, ranging from 0.005 to 1 mg/L by analyzing the standard solutions and the matrix-matched standard solutions with a coefficient of determination R ≥ 0.999. At two different spiked levels (0.01 mg/kg and 1 mg/kg) the recoveries of fluxapyroxad in apples amounted to 107.9 and 118.4%, and the RSDr were 4.2 and 4.7%. RSDwr was determined from on-going QC-data in routine analyses and it was 6.2%.

The LOD was defined on a basis of the noise level in the chromatograms at a signal-to-noise ratio (S/N) of 3:1, and the LOQ was defined as the lowest spiked level.

**Figure 2:**
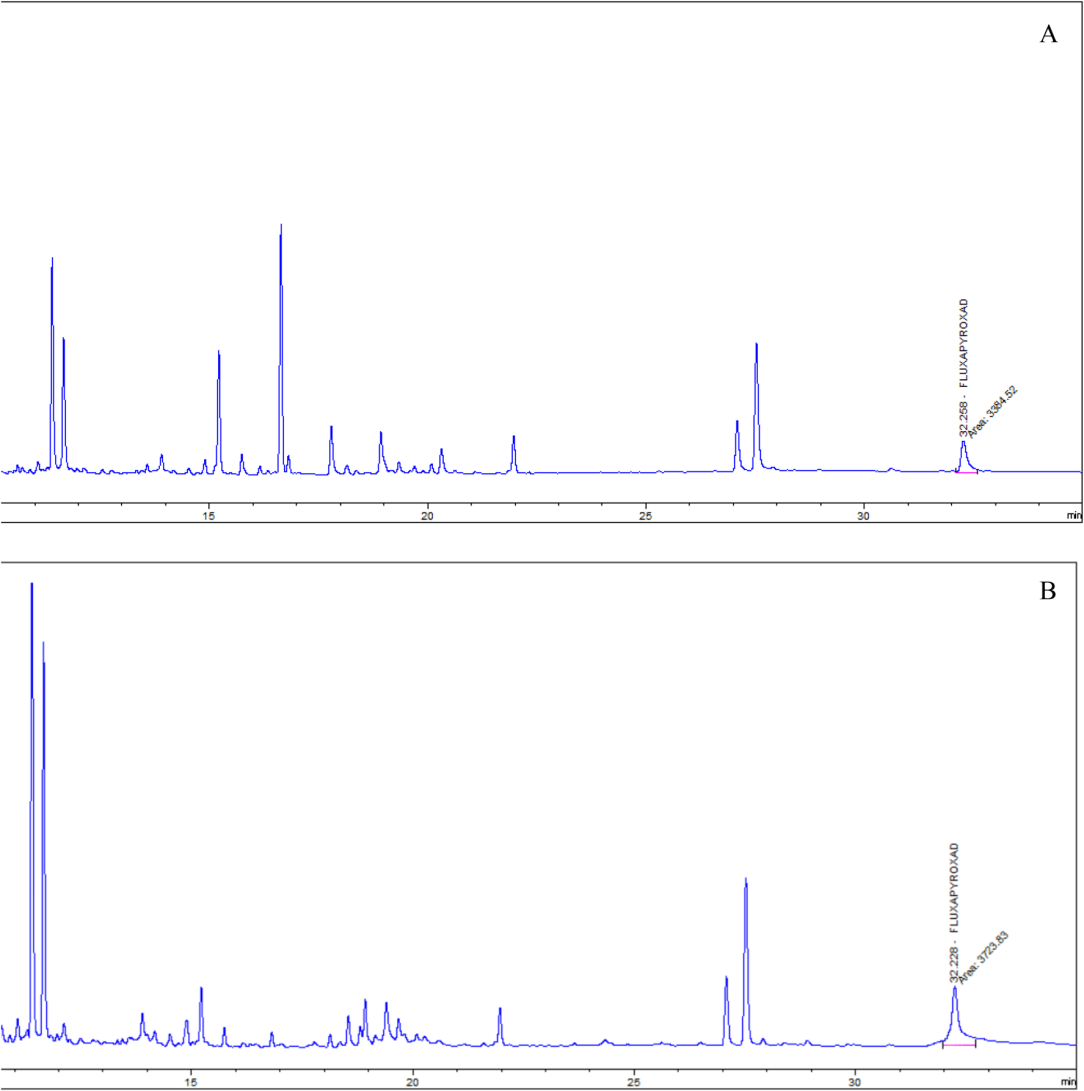
Response of fluxapyroxad in blank reagent (**A**) and blank matrix (**B**) at LOQ level.

Matrix effects were assessed by comparison of response from solvent standards and matrix-matched standards. Therefore, to compensate the matrix effects when identifying and quantifying the analytes, in this study matrix-matched standard solutions were used. Specificity of the method was determined by comparison response of fluxapyroxad in blank reagent and blank control samples (Fig. 2A,B). Robustness of the method was assessed by comparison of the average recovery and RSDwr, derived from on-going method validation. Retention time was determined by comparison response of fluxapyroxad in extract and standard solutions (with a tolerance of ± 0.1 min).

These results indicate that the parameters of this method met the analytical requirements of SANTE^14^. Therefore, it is a suitable method for analysing fluxapyroxad residues in apples.

### Dissipation of fluxapyroxad in apples

The treatment was conducted in the whole experimental plot, in the Gala variety, on June 17, 2018, and in the Idared on June 17, 2019. Sampling started on the next day (12 h after application), and continued after 1, 4, 8, 15, 29, 43, and 60 days. On the next day after treatment with Sercadis, fluxapyroxad initial residue levels were 0.417 ± 0.08 mg/kg in Gala, and, 0.304 ± 0.062 mg/kg in Idared (Table 1), and the concentration dissipated over the time, which confirms that after application the residues did not exceed MRL (0.9 mg/kg). The dissipation rates of 97.1% for Gala and 96.7% for Idared were recorded on 43 day after application, and they were below their LOD (0.005 mg/kg) on Day 60 after application.

**Table 1:** Sampling dates and fluxapyroxad residue levels after application of the preparations Sercadis i Myco-Sin in Gala and Idared varieties.

Sampling date	Number of days after chemical treatment	Fluxapyroxad concentration (mg/kg)	SD (mg/kg)	Fluxapyroxad concentration after treatment with Myco-Sin (mg/kg)	SD (mg/kg)
**Gala variety**					
6/18/2018	1	0.417	0.080	–	–
6/21/2018	4	0.291	0.064	–	–
6/25/2018	8	0.137	0.038	0.055	0.009
7/02/2018	15	0.051	0.018	0.039	0.013
7/16/2018	29	0.036	0.012	0.025	0.010
7/30/2018	43	0.012	0.003	0.011	0.005
8/16/2018	60	< LOD	–	< LOD	–
**Idared variety**					
6/18/2019	1	0.304	0.062	–	–
6/21/2019	4	0.285	0.052	–	–
6/25/2019	8	0.137	0.032	0.077	0.009
7/02/2019	15	0.091	0.030	0.067	0.020
7/16/2019	29	0.019	0.005	0.017	0.009
7/30/2019	43	0.010	0.004	< LOQ	0.007
8/16/2019	60	< LOD	–	< LOD	–

**Figure 3:**
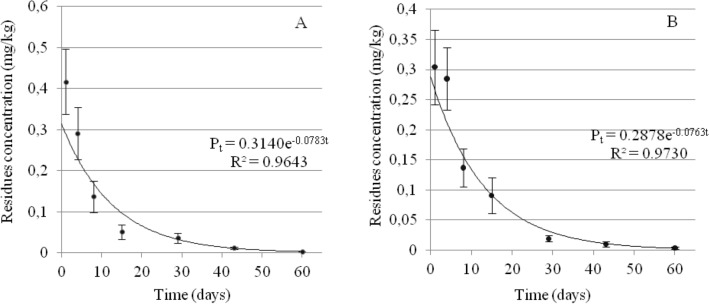
Dissipation trends for fluxapyroxad in apple samples after treatment with Sercadis in Gala (**A**) and Idared (**B**) varieties.

The dissipation of fluxapyroxad was determined by the first-order kinetics equations: P_t_ = 0.3140e^−0.0783t^ (R^2^ = 0.9643) and P_t_ = 0.2878e^−0.0763t^ (R = 0.9730) for Gala and Idared varieties, respectively (Fig. 3A,B). The corresponding half-life values were 8.9 and 9.0 days. According to current legislation, if ripe apples are to be used for production of foodstuffs for young children, the level of pesticide residues cannot exceed 0.01 mg/kg. On a basis of the above equations, the theoretical time of 44 days for fluxapyroxad dissipation to the concentration of 0.01 mg/kg was calculated for both varieties. In our experiment, the level of 0.01 mg/kg in the Idared variety was reached after 43 days.

Currently, fluxapyroxad is registered or being introduced worldwide for the use as the active substance of fungicides. It is necessary to monitor the amount of pesticides in the environment and to comply with relevant standards. Only a few studies have been conducted so far to assess the environmental fate, dispersion and degradation of this new fungicide. The Pesticide Properties Database reports that fluxapyroxad half-lives are: 183 days in soil under aerobic conditions, over 847 days in water–sediment, and 4.4 days in water phase^11^. The database does not provide the half-life of this active substance in plants. According to He et al.^12^ the half-lives of fluxapyroxad in apples and soil were 9.4–12.6 and 10.3–36.5 days, respectively. These authors performing trials using application rates of 78 g a.i. ha^−1^ (the recommended dose) and 118 g a.i. ha^−1^ (1.5 times the recommended dose) and performed treatments 2–3 times before harvesting. Noh et al.^2^ conducted research on the leaves of a perilla grown in a greenhouse (*Perilla frutescens var. Japonica Hara*)*.* Seven days after the application, the fluxapyroxad concentration decreased by 50.0% ± 4.9%. The dissipation kinetics of fluxapyroxad in soil were also studied. It was shown that the maximum t_1/2_ in soil was 157.6 days in aerobic conditions, and 345.4 days in anaerobic soil. The degradation in soil under anaerobic conditions was much slower than in aerobic conditions, which proves that aerobic microorganisms are more efficient in metabolizing fluxapyroxad^15^. To our knowledge, no other data concerning dissipation of fluxapyroxad in plants is available.

### Yeast influence on the fluxapyroxad degradation

**Figure 4:**
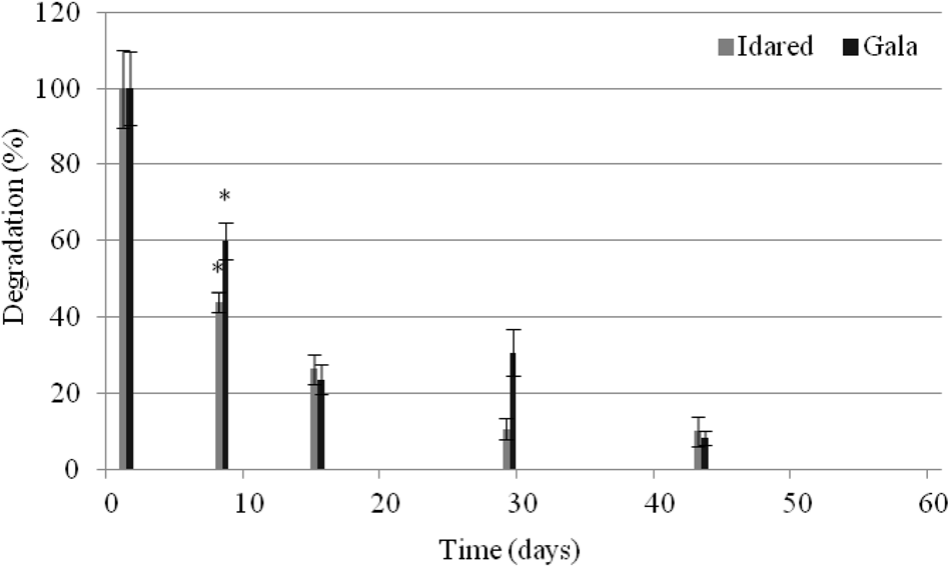
Fluxapyroxad degradation (%) after Myco-Sin treatment in Gala and Idared varieties.

The biological preparation Myco-Sin was applied in block II in Gala (June 24, 2018) and Idared (June 24, 2019) varieties. Samples treated with the biological preparation and control samples were collected on the next day (June 25), and then on the same dates as control samples. A day after the treatment with the biological preparation, the highest changes in fluxapyroxad concentration in apples in relation to the control samples were observed. The residues were determined at the levels of 0.055 ± 0.009 mg/kg and 0.077 ± 0.009 mg/kg, while in control samples residues were at levels 0.137 ± 0.038 mg/kg 0.137 ± 0.032 mg/kg in Gala and Idared varieties, respectively (Table 1). On the first day after Myco-Sin applications, the degradation rates were determined at the levels of 59.9% and 43.8% for Gala and Idared varieties, respectively (Fig. 4). On the subsequent days of the experiment, the degree of the fluxapyroxad degradation was 23.5%, and then of 30.6% and 8.3% in the Gala variety, and 23.4%, and then 10.5% and 10% in the Idared variety. On Day 60 after application, the residue levels in all samples were below their respective LODs (Fig. 4). Differences in fluxapyroxad concentrations in control and study samples were statistically significant only on Day 8 of sampling (p < 0.1).

A review of the literature on the degradation of pesticide residues indicates a great interest in the use of biological methods for degradation of active substances using bacteria and fungi. To date, literature data on yeasts and their effect on pesticide degradation is scarce. There are no studies on the fluxapyroxad degradation by yeasts.

The positive influence of yeasts on the acceleration of plant growth and protection against fungal pathogens is suggested^16^. A particular attention is paid to the species *S. cerevisiae*. These yeasts are particularly active in the rapid conversion of sugars into alcohol and carbon dioxide, thus contributing to the limited availability of nutrients for other organisms—plant pathogens. Additionally, they are capable of producing so-called ‘killer toxins’, protein complexes with a very strong inhibitory effect on pathogens found in the same environment^17,18^.

It has been proved that the yeast *S. cerevisiae* can be used for pesticide degradation. Wołejko et al.^18^ used this yeast species in their study on the degradation of strobilurins: azoxystrobin and pyraclostrobin, and carboxamides: iprodione and boscalid in lettuce leaves. The results showed that the addition of yeast to the tested substances accelerated the degradation of pesticide residues. In another study, the use of *S. cerevisiae* for the glyphosate degradation in the fermentation process of baking bread was analysed. It has been proven that these yeasts can degrade that pesticide by about 21% within 1 h^19–22^.

Kitagawa et al.^20^ stated that the introduction of the fungicide thiuram into the yeast environment of *S. cerevisiae* leads to changes in the gene expression in yeast cells. Many yeast genes responsible for detoxification were strongly induced. It was also established that the yeast *S. cerevisiae* is able to metabolize herbicides, atrazine and terbutylazine, to their hydroxyl derivatives in the beer production process^23^.

Another example of the yeasts used for the pesticide degradation is *Clavispora lusitaniae*, used to degrade pendimethalin in soil. This strain is able to degrade 74% of the active substance in 8 days of incubation^24^.

### Dietary risk assessment

The risk assessment for fluxapyroxad in apples was performed by estimation of %ADI, which was calculated by dividing the exposure by the ADI.

For Gala variety the estimated %ADI for general Polish consumer was 1.6%ADI and for Idared variety it was 1.4%ADI and is comparable with the same group form Germany (1.7–1.9%ADI) (Table 2). The obtained values of %ADI for the general Polish and German population are higher compared to the English adults. This is mainly due to lower apple consumption. Due to the lack of the data for Polish children we estimated the chronic exposure for German child and English infant and toddler. The highest values of 8.8–9.8%ADI for German children were assessed. For English infant and toddler the %ADI were ranged from 1.1 to 1.3 (Table 2). Those values (below 100%) indicating no risk of adverse effect following exposure to fluxapyroxad residues.

**Table 2:** Average intake, IEDI and %ADI of fluxapyroxad in different consumer groups.

Consumer group	Body weight (kg)	Average consumption (g/day)	Gala variety			Idared variety		
			STMR (mg/kg)	IEDI (g/kg bw)	%ADI	STMR (mg/kg)	IEDI (g/kg bw)	%ADI
Polish general	62.8	128.3	0.157	0.000321	1.6	0.141	0.000288	1.4
DE child	16.2	202.2		0.001959	9.8		0.001760	8.8
DE general	76.4	185.4		0.000381	1.9		0.000342	1.7
UK infant	8.7	13.6		0.000245	1.2		0.000220	1.1
UK toddler	14.6	24.9		0.000268	1.3		0.000240	1.2
UK adult	76.0	31.2		0.000064	0.3		0.000058	0.3

## Conclusions

In our study, the analytical method for determination of fluxapyroxad in apples was established. Fluxapyroxad residues in apples on the first day after treatment with Sercadis were much lower than the established MRL, and were 0.417 ± 0.08 mg/kg and 0.304 ± 0.082 mg/kg for Gala and Idared varieties, respectively. The dissipation half-lives of 9 days were calculated in two apple varieties, showing that fluxapyroxad disappears relatively quickly. To obtain the ripe apples intended for production of baby food with the pesticide residues level below 0.01 mg/kg, we established PHI of 44 days. In our studies, we also verified the influence of yeast on fluxapyroxad degradation in the field studies. Immediately after the application of the biological preparation Myco-Sin, the highest fluxapyroxad degradation levels of 59.9% for Gala and 43.8% for Idared were observed.

According to our results, the dietary risk is at the acceptable food safety level (below 9.8% for children, and 1.9% for adults) and does not pose a hazard to the consumer health.

## Material and methods

### Reagents and materials

The analytical standard fluxapyroxad (99.5% purity) was purchased from Supelco (Supelco Inc., Bellefonte, PA., USA)). HPLC-grade acetonitrile (ACN) was purchased from Honeywell (Charlotte, NC, USA), and petroleum ether (fraction boiling at 40–60 °C) of GC purity was supplied by Chempur (Piekary Śląskie, Poland). Sorbents: analytical grade MgSO_4_ and NaCitrate (Chempur, Piekary Śląskie, Poland), NaCl (Honeywell, Charlotte, NC, USA), disodium citrate sesquihydrate (Sigma Aldrich, Saint Louis, MO, USA), and primary-secondary amine (PSA) (Supelco Inc., Bellefonte, PA, USA) were used for QuEChERS extraction and purification.

Standard stock solutions of fluxapyroxad (1000 mg/L) were prepared in pure acetone, and were stored in the dark below − 20 °C. Standard working solutions of fluxapyroxad, at concentrations of 0.005, 0.01, 0.05, 0.1, 0.5, and 1 mg/L, were prepared from the stock solution by serial dilution. Correspondingly, matrix-matched standard solutions were obtained, at concentrations of 0.005, 0.01, 0.05, 0.1, 0.5, and 1 mg/L, by adding a blank sample matrix of apples to each serially diluted standard solution. All solutions were stored in a refrigerator in the dark at 4 °C. Apples for the preparation of a blank matrix were sampled from an organic orchard.

### Equipment, instruments and analytical conditions

The following equipment was used in our experiments: a vortex shaker (Bench Mixer™ BV 1000, Benchmark Scientific, Inc., Edison, NJ, USA), a centrifuge (5804R, Eppendorf, Hamburg, Germany), a homogenizer (Blixter 4, Robot Coupe, Vincennes, France), and a 100–1000 µL micropipette (Ependorf, Hamburg, Germany). An analysis of fluxapyroxad was performed on a 7890A gas chromatograph (Agilent Technologies, Santa Clara, CA, USA) equipped with a capillary column (HP-5 MS Ultra Inert/30 m × 0.25 mm I.D. × 0.25-μm) and a micro-electron capture detector (µECD). The following chromatographic parameters were used: sample injection in a splitless mode, an injected volume—2 μL, a carrier gas—helium (≥ 99.999% purity, a flow rate of 1.37 mL/min), and an auxiliary gas—nitrogen (≥ 99.999% purity, a flow rate of 40 mL/min). The inlet and detector temperatures were 250 °C and 300 °C, respectively. The initial column temperature was 100 °C, then it was increased to 180 °C at a rate of 10 °C per min and maintained for 4 min, and then it was again increased to 220 °C at a rate of 3 °C per min. Software ChemStation, Rev. B04.03-SP2 was used to process the data.

### Field experiments design

Field trials were conducted in 2018 and 2019 in two commercial apple orchards in Jozefów nad Wisłą in south-eastern Poland, Lubelskie voivodship, and in Rzeszów, in south-eastern Poland, Podkarpackie voivodship. The field trials were conducted in two varieties: Gala (Józefów nad Wisłą), and Idared (Rzeszów).

To protect apple trees against diseases, a chemical preparation Sercadis was used. Sercadis is the latest generation SDHI fungicide introduced into the market by BASF (Arnhem, Kingdom of the Netherlands) in 2017. It is used to prevent or combat diseases caused by fungi, *Venturia inaequalis* (apple scab) and *Podosphaera leucotricha* (powdery mildew of apples). This fungicide is distinguished from other pesticides by its flexible molecular structure, enabling even faster and more effective disease control. It has a form of a concentrated suspension, with a systemic mode of action. It contains an active substance fluxapyroxad at a concentration of 300 g/L. The recommended dose for a single application is in the range of 0.25–0.3 L/ha. Its PHI for apple is 35 days^25^.

A biological preparation used in our studies was Myco-Sin. It is an auxiliary preparation, increasing plant resistance. The active substances used in the preparation are aluminum sulfate tetradecahydrate (Al_2_H_28_O_26_S_3_, 740 g/kg), inactive, ground, dried yeast—*Saccharomyces cerevisiae* (140 g/kg of preparation), and dry horsetail extract (*Equisetum arvense* L.) (10 g/kg of preparation). Myco-Sin strengthens and changes pH on the leaf surface to alkaline. It is applied in crops, including fruit trees and shrubs by spraying, at a dose of 10 g/L of water for prevention of the infection spread. The PHI is 0 days, so the preparation can be used on a day of fruit harvesting^26^.

Apple trees of Gala and Idared varieties were sprayed on June 18, 2018 and June 18, 2019, respectively. The biological preparation Myco-Sin was applied 7 days after the treatment with the chemical preparation.

Degradation studies had a form of comparative experiments. Treatments with the chemical preparations were conducted in the whole experimental plot (about 0.5 ha) with a given variety, and the plot was later divided into two blocks, I and II, each containing six rows. Seven days after the treatments with the chemical preparation, in block I (control object) plants were sprayed with water, and in block II plants were sprayed with biological preparation.

**Figure 5:**
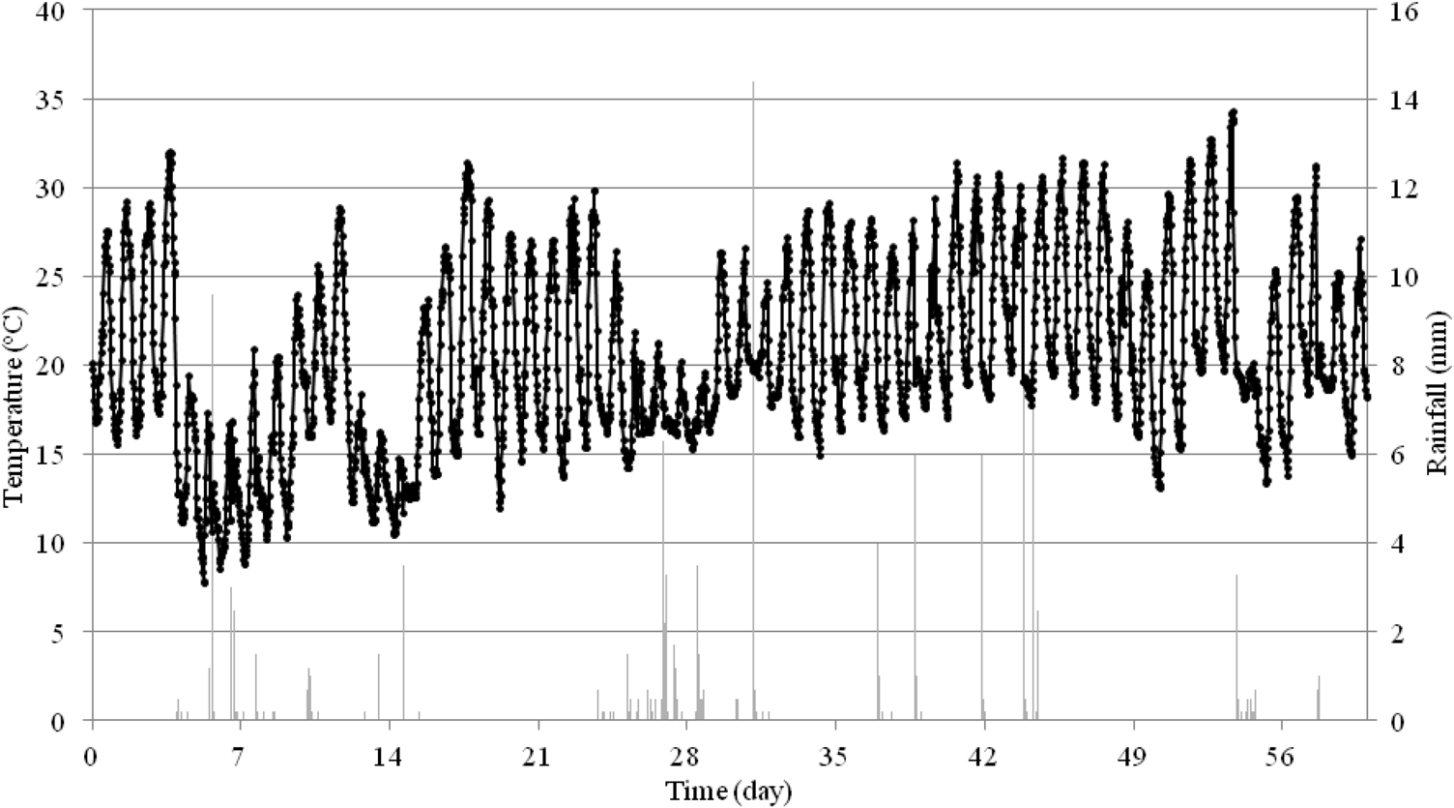
Temperature and precipitation in a period from 6/18/2018 to 8/16/2018 during the experiment in the Gala variety in Józefów nad Wisłą.

**Figure 6:**
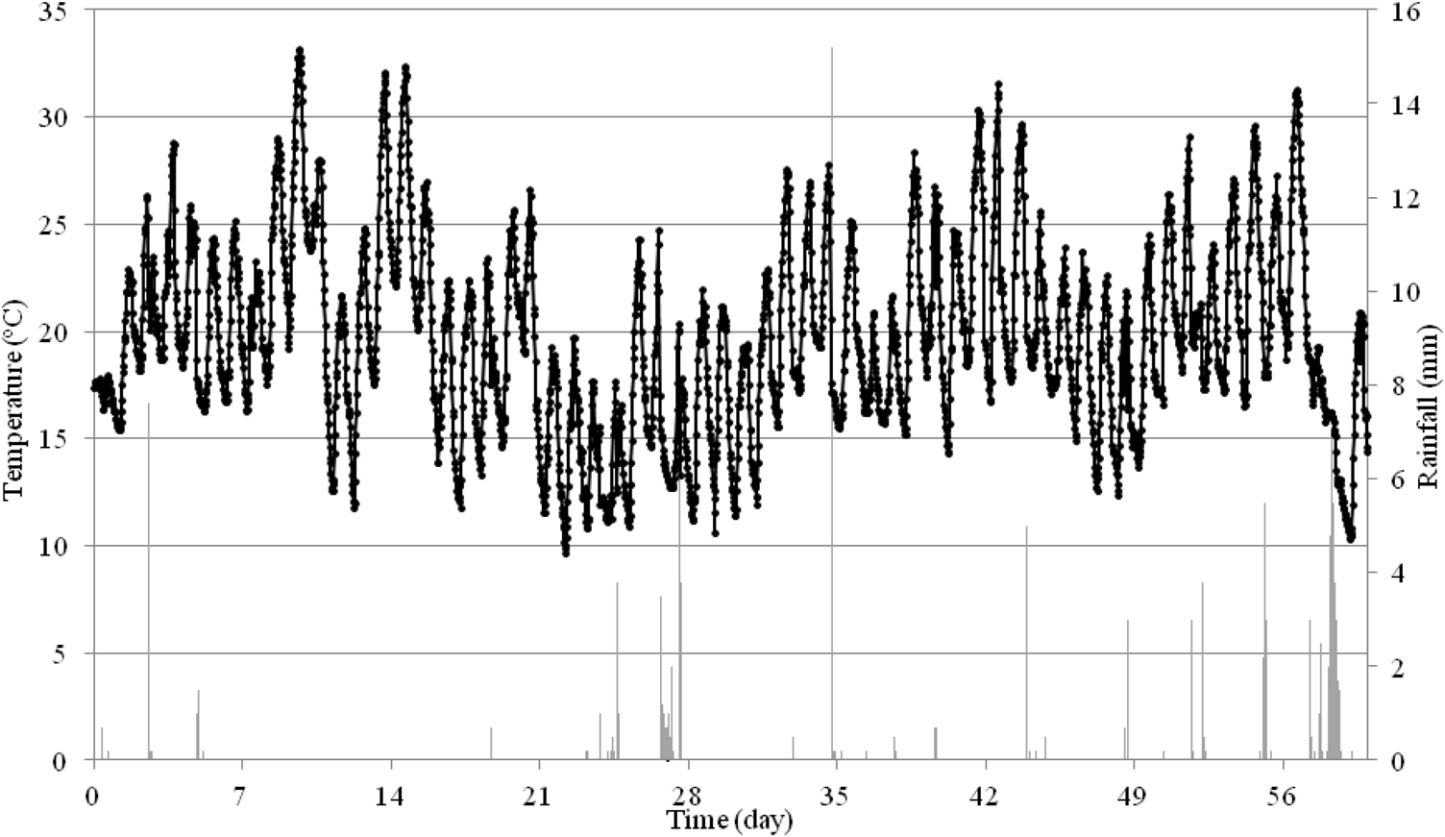
Temperature and precipitation in a period from 6/18/2019 to 8/16/2019 during the experiment in the Idared variety in Rzeszów.

Weather conditions in Józefów nad Wisłą and Rzeszów were measured using the WatchDog 2900ET weather station (Spectrum Technologies, Inc. Aurora, IL, USA) installed in the orchard. Temperatures (°C) and rainfall (mm) were recorded from the date of first spraying to the end of the trial (Figs. 5 and 6).

### Apple sampling

Laboratory samples of fruit were collected and processed in accordance with Regulation 2007 in four replications, from selected rows of trees^27^. On the first two sampling dates, four samples were taken from the entire experimental plot. After the treatment with the biological preparation, four apple samples were collected from each block, the control samples from block I and the test samples from block II.

The fruit samples were transported to the laboratory in clean containers, made of inert packaging materials, permanently and clearly marked. Approximately 1000–1500 g of apple samples, consisting of at least 10 units, were homogenized in a homogenizer, and then were mixed to ensure representativeness. Ten grams of analytical samples were weighted to 50 mL polypropylene centrifuge tubes with screw caps. The samples were stored in a freezer at − 17 °C until the day of extraction.

### Sample preparation

The analysis of fluxapyroxad in apples was done using a modified method based on the European Norm and literature on the QuEChERS method, involving sample extraction with ACN and clean-up through a dispersive solid phase extraction (d-SPE)^28^.

10 mL of ACN were added to the previously prepared analytical samples, and shaken vigorously for 1 min. Then buffer salts containing 4 g MgSO_4_, 1 g NaCl, 1 g Na_3_C_6_H_5_O_7_, and 0.5 g C_12_H_18_Na_4_O_17_ were added, the samples were shaken in a vortex for 1 min and then centrifuged at 3000 rpm for 5 min. Total of 5 mL of the organic phase were transferred to a 15-mL polypropylene centrifuge tube that contained sorbent for clean-up, consisting of 900 mg MgSO_4_ and 150 mg of PSA. Then the shaking and centrifugation step was repeated. The total of 750 µL of purified extract were transferred to a 2-mL chromatographic vial, the ACN extract was evaporated to dryness under a gentle stream of pure nitrogen and then dissolved in 750 µL of petroleum ether. The extracts were subjected to gas chromatography analysis with a µECD detector according to description in point 2.2.

The method was validated according to the SANTE recommendation^14^.

### Estimation of chronic exposure to fluxapyroxad

In food products, the consumer exposure to pesticide residues should be assessed in accordance with the recommendations published by the World Health Organization and taking into account the Community procedures and practices. The European Food Safety Authority (EFSA) introduced a revised model for calculating the consumer exposure—PRIMo rev. 3.1. The model was developed to calculate the dietary exposure to pesticide residues in food according to internationally agreed methodologies. In our paper chronic intake [expressed as International Estimated Daily Intake (IEDI), in mg/kg bw/day) of fluxapyroxad were assessed. The exposures were estimated and compared to the toxicological reference values (the Acceptable Daily Intake (ADI)]. Field studies did not show that fluxapyroxad concentrations were above MRL (0.9 mg/kg), therefore acute consumer exposure was not calculated.

For Polish consumer model PRIMo gives consumption data only for general Polish population, but the assessment of the dietary exposure should cover not only general population but also critical groups as children: infants and toddlers. Due to the lack of information for Polish children, we decided to compare the chronic exposure based on the consumption data from Germany and United Kingdom. Values of body weight and average consumption were taken from model PRIMo rev. 3.1.

The risk assessment of chronic intake of fluxapyroxad was calculated according to the following formula:

1 $$ {\text{IEDI}} = {{\left( {{\text{STMR}} \times {\text{F}}_{{\text{i}}} } \right)} \mathord{\left/ {\vphantom {{\left( {{\text{STMR}} \times {\text{F}}_{{\text{i}}} } \right)} {{\text{bw}}}}} \right. \kern-\nulldelimiterspace} {{\text{bw}}}} $$

2 $$ \% {\text{ADI}} = {\text{IEDI/ADI}} $$

where, STMR is the median fluxapyroxad residual from supervised trial (mg/kg), F_i_ is the food consumption data (g/day), bw is the body weight.

### Calculation and statistical analysis

The dissipation kinetics of fluxapyroxad were determined by plotting the residue concentration (mg/kg) versus the time elapsed after the treatment with the chemical preparation. This relationship was described by a kinetics equation for the first order reaction, according to the Eq. (3). The half-life was calculated according to Eq. (4)

3 $$ {\text{P}}_{{\text{t}}} = {\text{P}}_{0} \times {\text{e}}^{{ - {\text{kt}}}} $$

4 $$ {\text{t}}_{{{1}/{2}}} = {\text{ ln2}}/{\text{k}} $$

The theoretical residue dissipation time (t_0.01_) to 0.01 mg/kg was determined on a basis of the exponential equation:

5 $$ {\text{t}}_{{0.0{1}}} = {\text{ ln}}\left( {0.0{1}/{\text{P}}_{0} } \right)/\left( { - {\text{k}}} \right) $$

In these equations, P_t_ is the residue concentration (mg/kg) at the time t (days), P_0_ is the initial residue concentration (mg/kg), k is the first-order rate constant (expressed 1/day), and t_1/2_ is the time required for the pesticide residue level to decrease to the half initial concentration value after application (expressed in days).

The mean values ± standard deviation (SD) were calculated on a basis of four replicates during the dissipation and degradation with yeast processes. The statistical significance was assessed in Microsoft Office Excel, using Student's t-test for independent samples with a two-trace distribution. The analysed probability and variance values (p) < 0.1 were considered significant. The statistically significant differences were marked in the charts as: *p < 0.1.
